# New Perspectives in Health: Gut Microbiota

**DOI:** 10.3390/ijerph19105828

**Published:** 2022-05-10

**Authors:** Diana Cardona, Pablo Roman

**Affiliations:** 1 Department of Nursing, Physiotherapy and Medicine, University of Almeria, 04120 Almeria, Spain; pablo.roman@ual.es; 2 Health Research Center (CEINSA), University of Almeria, 04120 Almeria, Spain

The gut microbiota has an important role in different physiological functions, exerting effects from energy metabolism to psychiatric well-being. Several factors alter the gut microbiota, indeed altering the quality of health. The gut microbiota dysbiosis has been related to an increased susceptibility to intestinal, cardiovascular and nervous pathologies. This Special Issue focusing on “New perspectives in Health: Gut Microbiota” aims to cover recent and novel advancements, as well as future trends in the field of gut microbiota and health, given that more research is required to elucidate the role of microbiota, its outcomes in health, diseases and the pathways which are involved.

Topics addressed in this Special Issue include the influence of gut microbiota in several health diseases such as cancer, fibromyalgia or multiple sclerosis, as well as the development of new methods to analyze fecal samples or to detect probiotic bacterias, including different types of manuscripts such as clinical trials, reviews or observational studies. Twelve papers were published, covering different aspects of gut microbiota and health.

Two papers are clinical trials focused on the modulatory effects of probiotics in gut microbiota. The first one proved that a multispecies probiotic is effective to produce an improvement in attention in fibromyalgia [1]. The second one showed that combined strategy of a hypocaloric diet, percutaneous electrical stimulation and probiotics administration promoted a positive influence on anti-obesogenic gut bacteria by increasing muconutritive (*Akkermansia muciniphila*) and immunomodulatory (*Bifidobacterium* spp.) microbiota and Bacteroidetes phylum (*Prevotella* spp.) and reducing the ratio of *Firmicutes/Bacteroidetes* ratio [2]. On the topic of probiotics, a published systematic review in this Special Issue has shown that some probiotic strains (*Lactobacillus acidophilus*, *L. casei*, *Bifidobacterium longum*, or *L. rhamnosus*, among others) are an effective therapeutic strategy in some common treatment-related side effects in adult oncology patients [3]. In this context, it is important to elucidate which probiotic species has the best capability for withstand, thus, one study of this Special Issue compares five strains of probiotic (*Bifidobacterium* BB-12, *Lactobacillus rhamnosus* GG, *L. casei*, *L. acidophilus*, *L. plantarum*) and concludes that the *L. plantarum* strain had the best capability for growth [4].

Diet is an important modulator of gut microbiota so there are several papers in the Special Issue about this topic. One of them showed gut microbial and fatty acids changes after the ingestion of isoflavones, contributing to the understanding of the modulation of the gut microorganisms. Specifically, after isoflavone supplementation, the abundance of the genera *Slackia* significantly increased and the fecal microbial communities of menopausal women equol producers were more like no producers [5]. In fact, diet also plays an important role in multiple sclerosis, in which dysbiosis has been detected in relapsing–remitting patients receiving disease-modifying therapies. This gut microbiota alteration could be involved in increased intestinal permeability and affect clinical response and disease progression [6]. Related to the diet, the systematic review published about the effects of Titanium dioxide (TiO_2_) revealed that TiO_2_ alters the composition and the activity of intestinal bacteria. In addition, this food additive used in pastries, sweets and sauces, promoting an inflammatory environment in the gut and immune responses in animals with colitis or obesity [7]. In the same line, casomorphins from dairy and gliadorphin peptides from cereals produce intestinal inflammation and permeability, as well altered gut microbiota via opioid receptors, point to the importance to the vigilance to food-derived opioids [8].

One study protocol was also published in this Special Issue. This prospective study with 44 patients with Crohn’s disease with anti-TNFα treatment will provide additional evidence regarding potential non-invasive tools such as microbiota-based biomarkers to improve clinical management of these patients [9]. In this context, the development of new techniques of stool recollection such us a low-cost at-home stool collection kit for rural or low-resource settings are relevant [10].

Two review papers complete this Special Issue. The first one reviewed the relationship between nicotine receptors and gut microbiota, showing that the control of gut inflammation through α7 and α9 nAChRs, the vagus nerve, and the cholinergic anti-inflammatory pathway is fundamental [11]. The second one points to the relevance of gut microbiota in both osteoporosis (OP) and food allergy (FA). The disbyosis observed in these diseases causes the development of an important inflammatory substrate in the intestine, which leads to FA and the loss of estrogen typical of primary OP [12].

Overall, these 12 contributions published in this Special Issue further strengthen the essential function of gut microbiota in health and in various diseases. However, there are still many fundamental questions that remain unanswered, promising a great future for this field. Therefore, more research is necessary, and a second edition about this topic is proposed. Finally, the Guest Editors would like to sincerely thank all the authors for their valuable contributions.

## References

[1] Cardona D., Roman P., Cañadas F., Sánchez-Labraca N. (2021). The Effect of Multiprobiotics on Memory and Attention in Fibromyalgia: A Pilot Randomized Controlled Trial. Int. J. Environ. Res. Public Health.

[2] Lorenzo O., Crespo-Yanguas M., Hang T., Lumpuy-Castillo J., Hernández A.M., Llavero C., García-Alonso M.L., Ruiz-Tovar J. (2020). Addition of Probiotics to Anti-Obesity Therapy by Percutaneous Electrical Stimulation of Dermatome T6. A Pilot Study. Int. J. Environ. Res. Public Health.

[3] Rodriguez-Arrastia M., Martinez-Ortigosa A., Rueda-Ruzafa L., Ayora A.F., Ropero-Padilla C. (2021). Probiotic Supplements on Oncology Patients’ Treatment-Related Side Effects: A Systematic Review of Randomized Controlled Trials. Int. J. Environ. Res. Public Health.

[4] Stasiak-Różańska L., Berthold-Pluta A., Pluta A.S., Dasiewicz K., Garbowska M. (2021). Effect of Simulated Gastrointestinal Tract Conditions on Survivability of Probiotic Bacteria Present in Commercial Preparations. Int. J. Environ. Res. Public Health.

[5] Guadamuro L., Azcárate-Peril M.A., Tojo R., Mayo B., Delgado S. (2021). Impact of Dietary Isoflavone Supplementation on the Fecal Microbiota and Its Metabolites in Postmenopausal Women. Int. J. Environ. Res. Public Health.

[6] Pellizoni F.P., Leite A.Z., de Campos Rodrigues N., Ubaiz M.J., Gonzaga M.I., Takaoka N.N.C., Mariano V.S., Omori W.P., Pinheiro D.G., Matheucci Junior E. (2021). Detection of Dysbiosis and Increased Intestinal Permeability in Brazilian Patients with Relapsing-Remitting Multiple Sclerosis. Int. J. Environ. Res. Public Health.

[7] Rinninella E., Cintoni M., Raoul P., Mora V., Gasbarrini A., Mele M.C. (2021). Impact of Food Additive Titanium Dioxide on Gut Microbiota Composition, Microbiota-Associated Functions, and Gut Barrier: A Systematic Review of In Vivo Animal Studies. Int. J. Environ. Res. Public Health.

[8] Woodford K.B. (2021). Casomorphins and Gliadorphins Have Diverse Systemic Effects Spanning Gut, Brain and Internal Organs. Int. J. Environ. Res. Public Health.

[9] Sanchis-Artero L., Martínez-Blanch J.F., Manresa-Vera S., Cortés-Castell E., Rodriguez-Morales J., Cortés-Rizo X. (2020). Evaluation of Changes in Gut Microbiota in Patients with Crohn’s Disease after Anti-Tnfα Treatment: Prospective Multicenter Observational Study. Int. J. Environ. Res. Public Health.

[10] Fischer J.A.J., Karakochuk C.D. (2021). Feasibility of an At-Home Adult Stool Specimen Collection Method in Rural Cambodia. Int. J. Environ. Res. Public Health.

[11] Ruzafa L.R., Cedillo J.L., Hone A.J. (2021). Nicotinic Acetylcholine Receptor Involvement in Inflammatory Bowel Disease and Interactions with Gut Microbiota. Int. J. Environ. Res. Public Health.

[12] Sirufo M.M., De Pietro F., Catalogna A., Ginaldi L., De Martinis M. (2021). The Microbiota-Bone-Allergy Interplay. Int. J. Environ. Res. Public Health.

